# Diabetic Mastopathy: An Uncommon Complication of Diabetes Mellitus

**DOI:** 10.1155/2013/198502

**Published:** 2013-07-01

**Authors:** R. X. Kirby, D. I. Mitchell, N. P. Williams, D. A. Cornwall, S. O. Cawich

**Affiliations:** ^1^Department of Surgery, Radiology, Anaesthesia and Intensive Care, University of the West Indies, Mona, Kingston 7, Jamaica; ^2^Department of Pathology, University of the West Indies, Mona, Kingston 7, Jamaica; ^3^Section of Radiology, Department of Radiology, Anaesthesia, Surgery and Intensive Care, University of the West Indies, Mona, Kingston 7, Jamaica

## Abstract

*Introduction*. Whilst most consequences of diabetes mellitus are well recognized, breast-related complications remain obscure. The term diabetic mastopathy (DMP) attempts to describe the breast-related consequences of diabetes. *Methods*. We report the clinicopathologic findings in a patient with DMP and review the literature on this uncommon entity. *Results*. A 33-year-old woman with type 1 diabetes had excision biopsy of a 2 cm breast lump. Histopathologic evaluation revealed classic features of DMP: parenchymal fibrosis; keloid-like hyalinization of interlobular stroma; adipose tissue entrapment; lobular compression; dense chronic inflammatory cell infiltration; and lymphoid follicle formation. *Conclusion*. Clinicians should be aware of DMP as a differential for breast disease in women with uncontrolled diabetes.

## 1. Introduction

Whilst most consequences of diabetes mellitus are well recognized, breast-related complications remain obscure. The term diabetic mastopathy (DMP) attempts to describe one of the breast-related consequences of diabetes mellitus. To our knowledge, this is the first report of DMP encountered in the Anglophone Caribbean. 

## 2. Case Report

A 33-year-old woman with type I diabetes mellitus noted the presence of a nontender mass in the left breast that had been slowly increasing in size over a six-month period. Although she claimed compliance to insulin since being diagnosed with type I diabetes mellitus at age 15, there was objective evidence of poor endocrine control. Her history revealed a diagnosis of diabetic retinopathy and several HbA_1c_ values >8.0% in the 24-month period prior to her presentation. There were no high risk factors for breast cancer. 

Clinical examination revealed a 2 cm nontender lump in the upper outer quadrant of the left breast. The mass was firm, mobile, and not associated with any skin or nipple changes. There was no associated lymphadenopathy, and the contralateral breast was normal on palpation.

**Figure 1:**
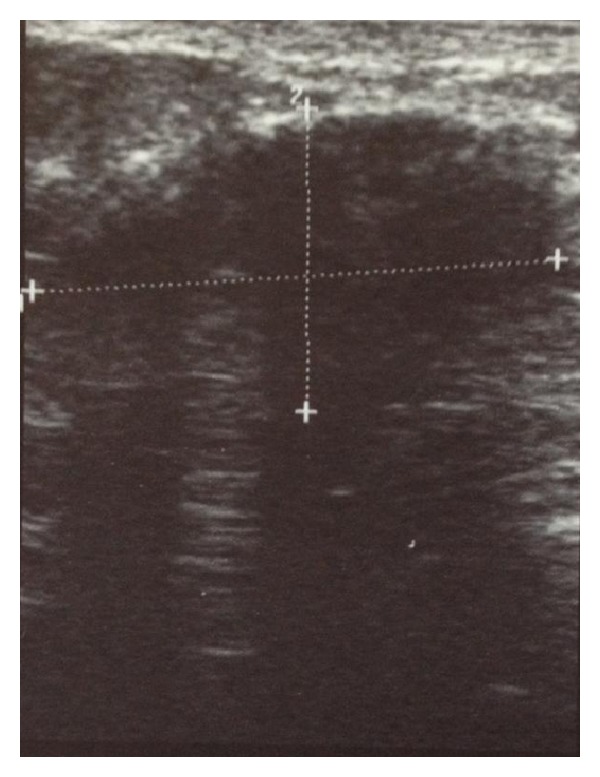
Ultrasonogram showing dense posterior acoustic shadowing of the lesion.

Breast ultrasonography revealed an ill-defined mass with strong posterior acoustic shadowing (Figure 1). Ultrasound guided core needle biopsy revealed no evidence of atypical cells but the yield was insufficient for diagnosis. Therefore, an excision biopsy was performed under local anaesthesia.

**Figure 2:**
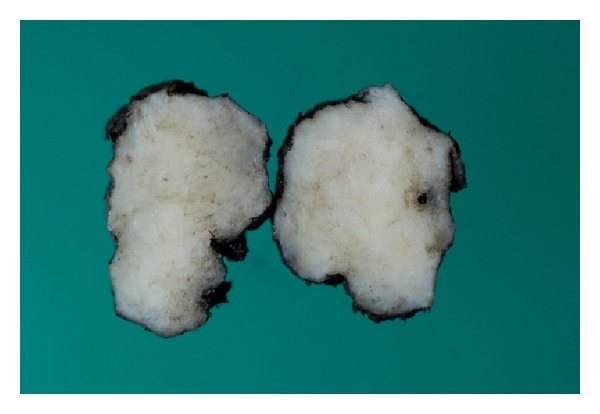
Excised breast mass cut to reveal the homogenous white fibrous parenchyma.

**Figure 3:**
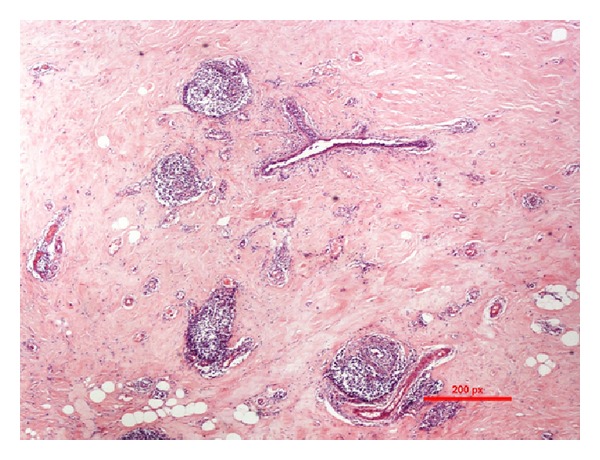
Microscopic images showing marked fibrosis and keloid-like hyalinization of interlobular stroma.

**Figure 4:**
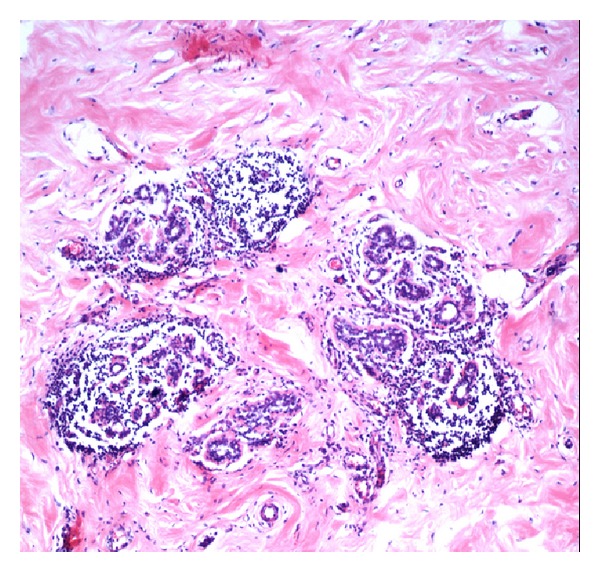
Microscopic images showing interlobular fibrosis with entrapped breast lobules and intralobular lymphocytic infiltration (H/E ×350).

**Figure 5:**
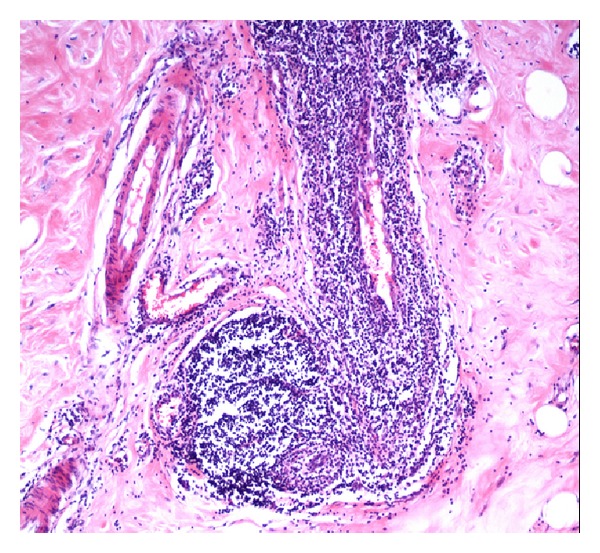
Microscopic images showing dense perivascular infiltrate with lymphoid follicle formation (H/E ×350).

The excised mass was firm and irregular with a solid white cut surface on gross inspection (Figure 2). Microscopic examination revealed breast tissue with no evidence of malignancy. However, there was marked fibrosis and keloid-like hyalinization of the interlobular stroma (Figure 3), with entrapment of adipose tissue and compression of the ducts and lobules (Figure 4). In addition, there was a dense chronic periductal and perilobular inflammatory cell infiltration, associated with lymphoid follicle formation (Figure 5). These features are pathognomonic for DMP. 

With a diagnosis of DMP, no further surgical management was deemed necessary. We continued regular breast surveillance with 6 monthly clinical evaluations and advised regular mammographic screening starting at the age of 40 years. At two years follow-up, there has been no recurrence of this lesion, and breast examinations have been normal to date.

## 3. Discussion

In 1984, Soler and Khardori [1] reported on a series of 12 women with longstanding type I diabetes mellitus and breast masses that mimicked cancer clinically but were benign on histological assessment. The term “diabetic mastopathy” was coined to describe these lesions, characterized by marked fibrosis and lymphocytic infiltrates [2].

To the best of our knowledge, this is the first report of DMP from the Caribbean region. It is an important differential to consider in young women with breast disease, especially in the Caribbean where the prevalence of diabetes mellitus is 15.7% among unselected women [3]. 

DMP has an incidence that ranges from 0.06% to 0.34% [4, 5] of benign breast lesions in unselected populations [4–6]. It has a weak association with type 2 diabetes and thyroid disease [1, 7–9] but a more robust relationship with insulin-dependent diabetes in premenopausal women [1, 4, 6]. The entity is diagnosed in 13% of these women [5], usually after living with type 1 diabetes for more than a decade [8, 9]. 

The majority of patients with DMP have poorly controlled diabetes. By the time breast disease manifests, between 75% and 80% [6, 8] of patients have already developed retinopathy, neuropathy, or nephropathy [1, 6, 8, 10]. This case was typical, occurring in a premenopausal woman, living with type 1 diabetes for 18 years with poor control as evidenced by the presence of diabetic retinopathy and serially elevated HBA_1c_ values.

Although the pathogenesis of DMP remains unsettled, the prevailing theories hold a prolific inflammatory response as the underlying pathophysiologic mechanism. There are several proposed inflammatory stimuli, including glycosylated native collagen in the breast parenchyma [7], deposition of glycosylated end products that act as neoantigens [6, 11], underlying autoimmune disease [1, 12], antibody cross-reactivity to exogenous insulin [13] or insulin contaminants [14], and altered tissue degradation due to locoregional cytokine imbalances [6, 7]. While debate continues about the inciting stimulus, the remaining pathophysiologic mechanisms are better understood. The chronic inflammatory process brings increased collagen deposition, potentiated collagen cross-linking, extracellular matrix expansion, and impaired cellular senescence. Simultaneously, there is infiltration of lymphocytes that drive the immune response, influx of macrophages, and deposition of other chronic inflammatory cells [6, 7].

The end result is a hard, irregular, nontender lump in diffusely thickened breast parenchyma no associated axillary lymphadenopathy [6, 15]. The lesions tend to be subareolar in location [8, 15, 16] and are bilateral in 20%–60% of cases [8, 16]. 

Imaging studies do not show any specific features of DMP [17]. However, there is usually a discrete mass with marked acoustic shadowing on ultrasonograms [4, 18] and homogenously dense glandular tissue without architectural distortion or microcalcification on mammograms [4]. Magnetic resonance imaging (MRI) may reveal nonspecific stromal enhancement without an enhancing mass. Although this differs from the enhancing focal masses with irregular margins that may be seen on MRI with carcinomas [19–21], the changes are not pathognomonic of DMP. There is usually increased focal uptake of  18-flourodeoxyglucose on PET-CT scans [22] but this is also a nonspecific finding. 

After thorough clinical and radiologic assessment, it is still difficult to differentiate DMP and breast carcinoma. The definitive diagnosis hinges on histologic assessment. Fine needle aspiration is not recommended because there is usually firm resistance to needle passes, making sampling difficult [16]. Moreover, there is insufficient cellular yield for diagnostic purposes in 50%–70% [4, 5] of cases. Core biopsy is the investigation of choice as it has better diagnostic yield than fine needle aspiration and avoids open biopsies [6, 15].

Histologic examination usually reveals firm, homogenous, white lesions that are indistinctly separated from surrounding normal breast tissue [23]. Microscopically, there is marked fibrosis with lymphocytes seen around the ducts, lobules, and vasculature [2]. Many of the fibroblasts undergo epithelioid change [6]. Although we note exceptions in few cases [8, 24], the findings generally considered to be pathognomonic for DMP include epithelioid fibroblasts set in a dense fibrous stroma as described by Tomaszewski et al. [7]; keloidal fibrosis (thick fibrous bands replacing >5 mm of parenchyma) as defined by Seidman et al. [9]; and the classic lymphocytic ductitis/lobulitis reported by Schwartz and Strauchen [11]. These pathognomonic microscopic features were all present in this case.

Traditionally, DMP has been considered a benign disease [4, 6, 8, 25, 26]. The four largest series in medical literature [4, 6, 8, 25] evaluated a combined cohort of 71 patients followed over a period of 6–14 years [4, 8]. There were no recorded instances of malignant transformation in these patients. Based on this data, many authorities recommend observation over surgical treatment since DMP is not considered a premalignant lesion [4, 6, 8, 15, 25, 26]. The recommendations were reinforced by the propensity for extensive local recurrence after excision biopsy in 42%–80% of cases within 5 years [6, 8]. Often the recurrences are bilateral, involving extensively more breast tissue than the original lesion. This may incite management dilemmas [27].

The medical literature contains four case reports documenting instances where DMP and ductal carcinoma coexist in the same breast [28–31]. This reinforces the need for exhaustive clinicopathological evaluation and a high index of suspicion. 

## 4. Conclusion

Diabetic mastopathy is an uncommon lesion, but clinicians should be aware of this entity in the Caribbean where there is a high prevalence of  diabetes mellitus.
